# Correction to: Intracellular vesicle-mediated biomineralization of arsenic and barium by a sponge symbiotic bacterium

**DOI:** 10.1093/ismeco/ycag224

**Published:** 2026-08-17

This is a correction to: Shani Shoham, Celeste Weiss, Ray Keren, Adi Lavy, Iryna Polishchuk, Boaz Pokroy, Abdussalam Azem, Micha Ilan, Intracellular vesicle-mediated biomineralization of arsenic and barium by a sponge symbiotic bacterium, *ISME Communications*, Volume 6, Issue 1, January 2026, ycag039, https://doi.org/10.1093/ismeco/ycag039

In three instances within the originally published version of this manuscript, “*Theonella swinhoei*” was erroneously replaced by “*Tamiops swinhoei*”.

These errors have been corrected within the ‘Abstract’, ‘Introduction’, and ‘Materials and Methods’ section.

